# Integrative Genomic and Functional Analysis Reveals NF1 Loss as a Modifier of DNA Damage and Replication Stress Responses in Ovarian Cancer

**DOI:** 10.1155/humu/9333284

**Published:** 2026-05-18

**Authors:** Shan He, Chengfeng Liu, Zhenyi Li, Tingjun Liao, Qin Wang

**Affiliations:** ^1^ Sichuan Provincial Center for Gynecology and Breast Diseases (Gynecology), Affiliated Hospital of Southwest Medical University, Luzhou, China, ahswmu.cn; ^2^ Department of Oncology, The Affiliated Traditional Chinese Medicine Hospital, Southwest Medical University, Luzhou, China, swmu.edu.cn; ^3^ Clinical Medical College, Southwest Medical University, Luzhou, China, swmu.edu.cn

**Keywords:** DNA damage repair, multiomics, NF1, NF1 mutation, ovarian cancer, replication stress

## Abstract

Neurofibromin 1 (NF1) is a tumor suppressor gene frequently altered across diverse cancer types, yet its biological significance in ovarian cancer remains incompletely characterized. Here, we integrated cohort‐scale somatic mutation profiling with functional validation to characterize the mutational and cellular consequences of NF1 loss in ovarian cancer. Somatic mutation data from the TCGA ovarian cancer cohort were analyzed to define NF1‐associated mutation types, tumor mutational burden, mutational signatures, and co‐occurring alterations in DNA damage repair (DDR) pathways, together with pathway‐ and gene set–level functional enrichment analyses. NF1 alterations were predominantly truncating and consistent with loss‐of‐function events. NF1‐mutant tumors did not exhibit increased global tumor mutational burden or uniform APOBEC hypermutation but showed distinct single‐nucleotide substitution patterns and frequent comutations in core DDR‐related genes. Functional enrichment analyses further highlighted coordinated involvement of pathways related to DNA replication, RNA processing, and proteostasis. Clinically, NF1 mutation was not independently associated with overall survival. Stable NF1 knockdown ovarian cancer models showed that NF1 depletion did not affect basal proliferation but increased sensitivity to hydroxyurea‐induced replication stress, accompanied by increased *γ*H2AX accumulation. Together, these findings indicate that NF1 loss defines a DNA damage–associated mutational and cellular state in ovarian cancer. Rather than acting as a direct prognostic determinant, NF1 mutation appears to increase vulnerability to replication stress and DNA damage, providing functional insight into its role in ovarian tumor biology.

## 1. Introduction

Ovarian cancer remains a globally lethal gynecologic malignancy, with an estimated 324,603 new cases and 206,956 deaths worldwide in 2022, underscoring a persistently unfavorable mortality burden [1]. In the United States alone, ~21,010 women are expected to be newly diagnosed and ~12,450 to die from ovarian cancer in 2026 [2]. Although therapeutic advances have improved outcomes for selected molecular subsets, population‐level survival is still constrained; for invasive epithelial ovarian cancer, the 5‐year relative survival rate is ~51% across SEER stages [3, 4]. This clinical reality reflects profound intertumor genomic heterogeneity and the coexistence of distinct mutational processes that shape tumor evolution, therapeutic vulnerabilities, and resistance trajectories [5–7]. Consequently, delineating mutation‐defined subgroups and linking their mutational signatures to pathway‐level liabilities has become central to precision oncology in ovarian cancer, providing a rational framework to nominate clinically actionable biomarkers and potentially mechanistically grounded targets [8–10].

NF1 encodes neurofibromin, a multifunctional tumor suppressor with roles in RAS pathway regulation and broader cellular homeostasis [11, 12]. Somatic NF1 alterations are observed across multiple malignancies and often present as truncating, loss‐of‐function events [13, 14]. However, the biological meaning of NF1 mutation is context dependent: in some tumor types it associates with distinct mutational architectures, co‐occurring genome instability programs, and altered stress responses, whereas its prognostic value can be weak or inconsistent [15, 16]. In ovarian cancer, NF1 mutations have been reported but remain comparatively underexplored, and it is unclear whether NF1 loss merely reflects background genomic instability or instead defines a coherent mutational context with functional consequences [17–19]. This gap is particularly relevant because ovarian tumors can exhibit heterogeneous mutagenic processes—including APOBEC‐associated cytidine deamination—and variable DDR disruption, both of which influence replication dynamics and DNA damage burden [20–23].

A central challenge is therefore to move beyond frequency‐based descriptions and determine whether NF1 loss anchors a reproducible genomic state in ovarian cancer. Specifically, three questions remain unresolved: (i) What is the mutation type spectrum of NF1 alterations and how do NF1‐mutant tumors differ in global mutational features such as tumor mutational burden (TMB) and substitution patterns; (ii) whether NF1 mutation is associated with coordinated DDR coalteration frameworks that could rationalize downstream vulnerability to replication perturbation; and (iii) whether these genomic associations translate into measurable functional phenotypes in ovarian cancer cells, particularly under replication stress where DDR capacity becomes rate limiting.

Here, we integrate cohort‐scale somatic mutation profiling from the TCGA ovarian cancer dataset with targeted in vitro validation to characterize NF1‐associated tumor states. We systematically define the global mutational landscape, delineate NF1‐linked mutational processes and DDR comutation patterns, and interpret downstream functional consequences through pathway‐ and gene set–based analyses. Finally, using stable NF1 knockdown models, we test whether NF1 deficiency alters basal proliferation or instead selectively compromises cellular responses to replication stress and promotes DNA damage accumulation. Collectively, this work positions NF1 loss as a modifier of mutational architecture and replication stress vulnerability in ovarian cancer, providing a mechanistic framework that connects tumor genomic context with stress‐contingent cellular phenotypes.

## 2. Methods

### 2.1. Somatic Mutation Data Acquisition and Global Mutational Profiling

Somatic mutation data for ovarian cancer were obtained from the TCGA‐OV cohort in Mutation Annotation Format (MAF) [24, 25]. High‐confidence single‐nucleotide variants and small insertions/deletions were retained after standard quality control. Gene‐level mutation frequencies, mutation type distributions, and individual mutation events were summarized using established somatic mutation analysis frameworks [26]. TMB was calculated as the number of nonsynonymous mutations per megabase and visualized on a log‐transformed scale [27, 28]. APOBEC‐associated mutational enrichment was quantified based on trinucleotide substitution patterns to capture intertumor heterogeneity in cytidine deaminase–mediated mutagenesis [29].

### 2.2. Inference of Candidate Driver Genes

Candidate driver genes were inferred using mutation pattern–based statistical models that evaluate deviations from expected background mutation rates and positional clustering of variants within coding regions [30, 31]. Genes showing significant evidence of positive selection were designated as inferred candidate drivers, emphasizing computational inference rather than prior biological annotation [32].

### 2.3. Definition of NF1 Mutation Status and Subgroup Stratification

NF1 mutations were extracted from the TCGA‐OV MAF dataset and categorized according to predicted functional consequence. Tumors were subsequently classified as NF1‐mutant or NF1–wild‐type based on the presence of at least one high‐confidence nonsynonymous or truncating NF1 alteration. This binary stratification was used consistently across all downstream analyses.

### 2.4. NF1‐Associated Mutational Features and APOBEC Activity

TMB and APOBEC‐associated mutational enrichment were compared between NF1‐mutant and NF1–wild‐type tumors. APOBEC activity was analyzed both as a continuous enrichment score and as a categorical APOBEC‐enriched versus nonenriched classification based on established thresholds as previously described. In addition, single‐nucleotide variants were summarized into six substitution classes to characterize NF1‐associated differences in mutational spectra.

### 2.5. DNA Damage Repair (DDR) Gene Sets and NF1‐Centered Comutation Analysis

DDR gene sets were curated from established pathway annotations, encompassing homologous recombination (HR), Fanconi anemia (FA), mismatch repair, nucleotide excision repair, and related processes [33, 34]. For each tumor, pathway‐level alteration status was defined by the presence of at least one nonsynonymous mutation within the corresponding gene set. Gene‐level and pathway‐level DDR mutation burdens were quantified as the number of nonsynonymous mutations and compared between NF1‐mutant and NF1‐wild‐type tumors. NF1‐centered comutation patterns were further evaluated within NF1‐mutant tumors to identify preferentially coaltered DDR genes.

### 2.6. Clinical Data Acquisition and Survival Analysis

Clinical annotations were retrieved from harmonized TCGA clinical files [35]. Overall survival was defined as the interval from diagnosis to death or last follow‐up, with censoring applied to patients alive at last contact. Kaplan–Meier survival curves were compared using log‐rank tests [36, 37]. Multivariable Cox proportional hazards models were fitted to evaluate the independent prognostic contribution of NF1 mutation status or APOBEC enrichment after adjustment for age at diagnosis [38].

### 2.7. Mutation Effect– and Gene Set–Based Functional Analyses

Mutation effect–oriented pathway analysis was performed using an integrative cancer genomics platform to evaluate the functional impact of NF1‐associated mutations at the pathway level in ovarian cancer [39, 40]. In parallel, gene set enrichment analysis was conducted using a curated gene set representing proliferation, replication, RNA processing [41], and metabolic processes to assess coordinated functional patterns associated with NF1 alteration status.

### 2.8. Cell Lines and Construction of Stable Knockdown Cell Lines

Human ovarian cancer cell lines SK‐OV‐3 (RRID: CVCL_0532), OVCAR‐3 (RRID: CVCL_0465), and HEK293T packaging cells (RRID: CVCL_0063) were kindly provided by the Central Laboratory of the Affiliated Hospital of Southwest Medical University. SK‐OV‐3 and OVCAR‐3 cells were cultured in RPMI‐1640 medium (Gibco) supplemented with 10% fetal bovine serum (FBS, Gibco) and 1% penicillin‐streptomycin (Gibco), whereas HEK293T cells were maintained in DMEM (Gibco) with 10% FBS. All cells were cultured at 37°C in a humidified incubator containing 5% CO_2_.

Two short hairpin RNAs targeting NF1 (shNF1‐1 and shNF1‐2) were cloned into the pLKO.1‐puro lentiviral vector. Lentiviral particles were produced by cotransfecting HEK293T cells with the recombinant pLKO.1‐shNF1 plasmids and the packaging plasmids psPAX2 and pMD2.G using Lipofectamine 3000 (Thermo Fisher). Viral supernatants were collected 48 h posttransfection, filtered through 0.45 *μ*m filters, and used to infect SK‐OV‐3 and OVCAR‐3 cells. After 24 h, puromycin (2 *μ*g/mL, Sigma) was added for 5–7 days to select stable knockdown cell lines. Knockdown efficiency was assessed by qRT‐PCR, and the shRNAs were used for subsequent experiments.

### 2.9. Cell Counting Kit‐8 (CCK‐8) Cell Viability Assay

SK‐OV‐3 and OVCAR‐3 cells stably expressing shNC, shNF1‐1, or shNF1‐2 were seeded in 96‐well plates at a density of approximately 3000 cells per well. Cell viability was assessed at 0, 24, 48, 72, and 96 h using the CCK‐8 (CCK‐8, Biosharp, BS350A). At each time point, 10% CCK‐8 solution was added to each well and incubated at 37°C for 1 h, followed by measurement of optical density (OD) at 450 nm using a microplate reader. Each group was measured in triplicate, and experiments were repeated three times. Growth curves were plotted based on average OD values.

To assess the effect of NF1 knockdown on sensitivity to replication stress, cells were treated with hydroxyurea (HU, 0.25 mM, MCE) 24 h after seeding. Cells were then cultured for an additional 72 h, and OD values were recorded at 48, 72, and 96 h using the same CCK‐8 protocol. Growth inhibition was compared between groups to evaluate HU‐induced sensitivity. All measurements were performed in triplicate and repeated in at least three independent experiments. Data were used to generate growth curves and for statistical analysis.

### 2.10. Quantitative Real‐Time PCR

Total RNA was extracted from cells using an RNA isolation kit (EZBioscience, B0004D), and the concentration and purity were determined using a NanoDrop 2000 spectrophotometer. Equal amounts of RNA were reverse‐transcribed into cDNA using HiScript III RT SuperMix (Vazyme, R323‐01). Quantitative real‐time PCR was performed using ChamQ SYBR qPCR Master Mix (Vazyme, Q311‐02) in a 20‐*μ*L reaction volume on a QuantStudio 5 Real‐Time PCR System (Thermo Fisher).

The thermal cycling conditions were as follows: initial denaturation at 95°C for 30 s, followed by 40 cycles of 95°C for 10 s and 60°C for 30 s. All reactions were run in triplicate. GAPDH was used as the internal control, and relative gene expression was calculated using the 2^−*ΔΔ*Ct^ method. All primers were synthesized by Tsingke Biotechnology (Sichuan, China). Primer sequences are listed below:

• GAPDH‐F: GACAGTCAGCCGCATCTTCT.
• GAPDH‐R: GCGCCCAATACGACCAAATC.
• NF1‐F: AAAACCAGCGGAACCTCCTT.
• NF1‐R: GCTGGCTAACCACCTGGTATAAA.

### 2.11. Flow Cytometric Analysis of *γ*H2AX

To evaluate DNA damage accumulation under replication stress, flow cytometry was used to quantify *γ*H2AX expression levels. SK‐OV‐3 and OVCAR‐3 cells stably expressing shNC, shNF1‐1, or shNF1‐2 were seeded in 6‐well plates. After 24 h, cells were treated with HU (0.25 mM, MCE) for 24 h to induce replication stress.

Cells were harvested, washed with PBS, and fixed with 1% paraformaldehyde (PFA) for 10 min, followed by overnight fixation in prechilled 70% ethanol at 4°C. After washing with PBS, cells were permeabilized with 0.2% Triton X‐100 for 10 min. After blocking, cells were incubated with anti‐*γ*H2AX primary antibody (Cell Signaling Technology, #9718, 1:200) at room temperature for 1 h in the dark. After washing, Alexa Fluor 488–conjugated goat anti‐rabbit IgG secondary antibody (Invitrogen, 1:1000) was added and incubated for 30 min in the dark. Finally, cells were washed and resuspended in PBS for flow cytometry.

At least 10,000 events per sample were acquired using a BD FACSCalibur flow cytometer and analyzed with FlowJo software. Debris and doublets were excluded by appropriate gating. The geometric mean fluorescence intensity (gMFI) of *γ*H2AX was normalized to the shNC group (set as 100%) and expressed as relative fluorescence intensity (%) for comparison of DNA damage levels across groups.

### 2.12. Statistical Analysis

All statistical analyses were performed using R software (Version 4.5.1). Continuous variables were summarized as means ± standard deviation or medians with interquartile ranges, as appropriate. Comparisons between two groups were conducted using two‐sided Wilcoxon rank‐sum tests for nonnormally distributed variables or unpaired two‐tailed Student′s *t* tests for experimental data. Categorical variables were compared using Fisher′s exact test. Survival probabilities were estimated using the Kaplan–Meier method and compared by log‐rank tests, and multivariable survival analyses were performed using Cox proportional hazards regression models with age at diagnosis included as a covariate. All statistical tests were two‐sided, and a *p* value < 0.05 was considered statistically significant unless otherwise stated.

## 3. Result

### 3.1. Global Mutational Features Define the Genomic Background of Ovarian Cancer

**Figure 1:**
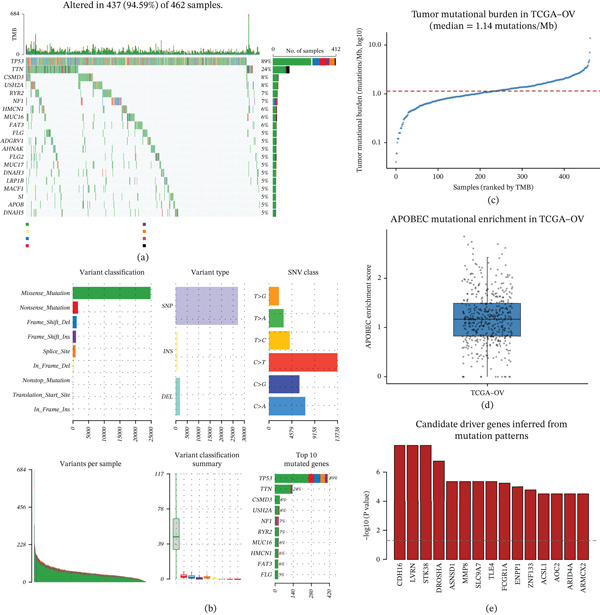
Global mutational landscape and inferred driver architecture of ovarian cancer. (a) Oncoplot showing the mutation landscape of the most frequently altered genes across the TCGA‐OV cohort, with samples arranged according to mutational profiles and gene alteration frequencies indicated. (b) Summary of mutation classifications and mutation types, showing the predominance of missense mutations, the dominance of single‐nucleotide polymorphisms (SNPs), and the prevalence of C > T substitutions among SNV classes. (c) Tumor mutational burden (TMB) across ovarian cancer samples, presented on a log scale, with the median TMB indicated. (d) Distribution of APOBEC‐associated mutational enrichment scores, demonstrating heterogeneity in APOBEC‐mediated mutagenesis among tumors. (e) Inferred candidate driver genes identified using mutation pattern–based statistical analyses, highlighting genes whose mutation patterns deviate from background expectations.

To establish the global mutational context of ovarian cancer, we first profiled the somatic mutation landscape of the TCGA‐OV cohort. The oncoplot showed that 437 of 462 tumors (94.59%) harbored detectable somatic alterations, with TP53 representing the dominant recurrently mutated gene, whereas other alterations occurred at substantially lower frequencies, indicating marked interpatient heterogeneity beyond the TP53‐centered mutational backbone (Figure 1a). Variant classification analysis showed that missense mutations were the predominant alteration category, whereas nonsense and frameshift events were present at lower frequencies; at the variant‐type level, single‐nucleotide polymorphisms (SNPs) constituted the major mutation type (Figure 1b). Analysis of substitution classes further showed that C > T transitions were the most prevalent SNV category in this cohort (Figure 1b). TMB displayed a right‐skewed distribution with a relatively low median value of 1.14 mutations/Mb, indicating an overall modest mutation burden across TCGA‐OV samples (Figure 1c). In parallel, APOBEC‐associated mutational enrichment scores exhibited substantial intertumor variability, suggesting heterogeneous engagement of APOBEC‐related mutagenic processes within the cohort (Figure 1d). Using mutation pattern–based inference, we further identified a panel of candidate driver genes inferred from mutation patterns, highlighting genes whose mutational features significantly deviated from background expectations (Figure 1e). Together, these analyses delineate the baseline mutational architecture of ovarian cancer and provide a genomic framework for subsequent NF1‐centered analyses.

### 3.2. NF1 Loss‐of‐Function Alterations Couple to a Distinct Mutational Backdrop Without Globally Elevated Mutation Burden

**Figure 2:**
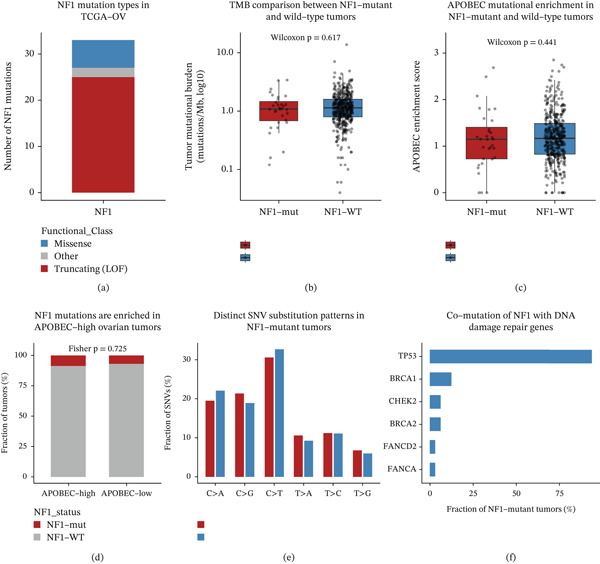
NF1 mutation defines a distinct mutational context in ovarian cancer. (a) Distribution of NF1 mutation types in the TCGA‐OV cohort, showing that truncating loss‐of‐function alterations predominate over missense and other mutation classes. (b) Comparison of tumor mutational burden (TMB) between NF1‐mutant and NF1‐wild‐type tumors, showing no significant difference between groups. (c) APOBEC enrichment scores stratified by NF1 mutation status, indicating comparable APOBEC‐associated mutagenic activity between NF1‐mutant and wild‐type tumors. (d) Proportion of NF1‐mutant and NF1‐wild‐type tumors across APOBEC‐high and APOBEC‐low groups, showing no significant association between NF1 mutation and categorical APOBEC enrichment status. (e) Six‐class single‐nucleotide variant (SNV) substitution spectra in NF1‐mutant and NF1‐wild‐type tumors, showing differences in mutational composition between groups. (f) Comutation profile of NF1‐mutant tumors, highlighting frequent concurrent alterations in TP53 and several DDR‐related genes, including BRCA1, BRCA2, CHEK2, FANCD2, and FANCA.

To move from the global mutational landscape to a gene‐centered analysis, we next examined the mutational features associated with NF1 in ovarian cancer. NF1 alterations were predominantly truncating, consistent with loss‐of‐function inactivation (Figure 2a). Despite this clear loss‐of‐function pattern, NF1‐mutant tumors did not show a significantly higher TMB than NF1‐wild‐type tumors (Wilcoxon *p* = 0.617), indicating that NF1 mutation is not simply associated with global hypermutation (Figure 2b). Similarly, APOBEC enrichment scores were comparable between the two groups (Wilcoxon *p* = 0.441), suggesting no evident association between NF1 mutation and cohort‐wide APOBEC activity when assessed as a continuous variable (Figure 2c). Consistently, NF1 mutation status was not significantly associated with APOBEC‐high versus APOBEC‐low classification (Fisher *p* = 0.725) (Figure 2d). Comparative analysis of six‐class SNV substitution spectra nevertheless showed differences in mutational composition between NF1‐mutant and NF1–wild‐type tumors, suggesting that NF1 alteration may be linked to a distinct mutational process context even in the absence of marked differences in global TMB or APOBEC enrichment (Figure 2e). In addition, comutation profiling within NF1‐mutant tumors showed frequent concurrent alterations in TP53 and several DDR‐related genes, including BRCA1, BRCA2, CHEK2, FANCD2, and FANCA, placing NF1 loss within a broader genome maintenance–related mutational context rather than as an isolated event (Figure 2f). Together, these results indicate that NF1 loss‐of‐function mutations define a distinct mutational backdrop characterized by altered substitution patterns and recurrent coalterations in genome stability–related genes, without manifesting as globally elevated TMB or uniform APOBEC enrichment.

### 3.3. NF1 Mutation Is Associated With Coordinated Alterations in DNA Damage Repair Pathways

**Figure 3:**
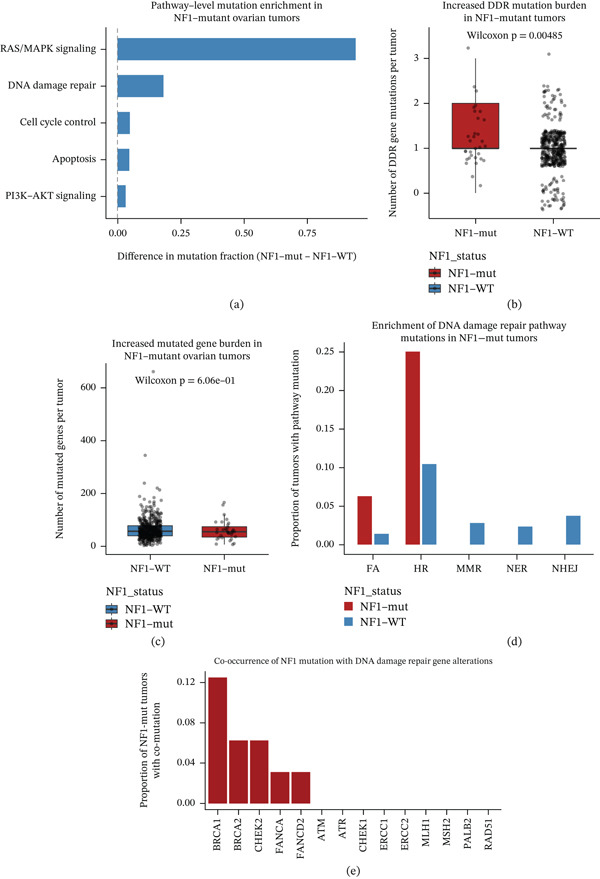
NF1‐centered DNA damage repair alteration landscape in ovarian cancer. Pathway‐ and gene‐level analyses delineating the association between NF1 mutation and DNA damage repair (DDR) alterations in the TCGA‐OV cohort. (a) Comparison of DDR pathway mutation frequencies between NF1‐mutant and NF1–wild‐type tumors, highlighting enrichment of homologous recombination and Fanconi anemia pathways in NF1‐mutant cases. (b) Overall DDR pathway mutation burden stratified by NF1 status. (c) Gene‐level DDR mutation burden comparing NF1‐mutant and NF1‐wild‐type tumors. (d) Functional enrichment analysis identifying DDR pathways preferentially affected in NF1‐mutant tumors. (e) NF1‐centered comutation landscape illustrating the proportion of NF1‐mutant tumors harboring concurrent alterations in core DDR genes, including BRCA1, BRCA2, CHEK2, and FANCD2.

To investigate whether NF1 mutation delineates a distinct genomic context beyond overall mutational burden, we performed pathway‐ and gene‐level analyses focusing on DDR processes in the TCGA‐OV cohort. Pathway‐based enrichment analysis revealed that NF1‐mutant tumors exhibited a higher prevalence of alterations across multiple DDR pathways, with particularly pronounced enrichment in HR and FA–related genes compared with NF1‐wild‐type counterparts (Figure 3a). Consistent with this observation, NF1‐mutant tumors showed an increased burden of DDR‐related mutations at both the pathway and gene levels, indicating coordinated disruption rather than isolated gene‐specific events (Figure 3b,c). Functional enrichment analysis further confirmed that DDR pathways were among the most significantly overrepresented mutational programs in NF1‐mutant tumors (Figure 3d). Notably, comutation analysis demonstrated preferential co‐occurrence between NF1 and core DDR genes, including BRCA1, BRCA2, CHEK2, and FANCD2, highlighting a structured DDR alteration landscape rather than random mutational accumulation (Figure 3e). Collectively, these results position NF1 mutation as a genomic stratifier associated with a DDR‐oriented mutational architecture in ovarian cancer, providing a mechanistic link between NF1 status and downstream genomic instability phenotypes.

### 3.4. NF1 Mutation Is Not an Independent Prognostic Determinant But Stratifies Genomic Context Without Survival Impact

**Figure 4:**
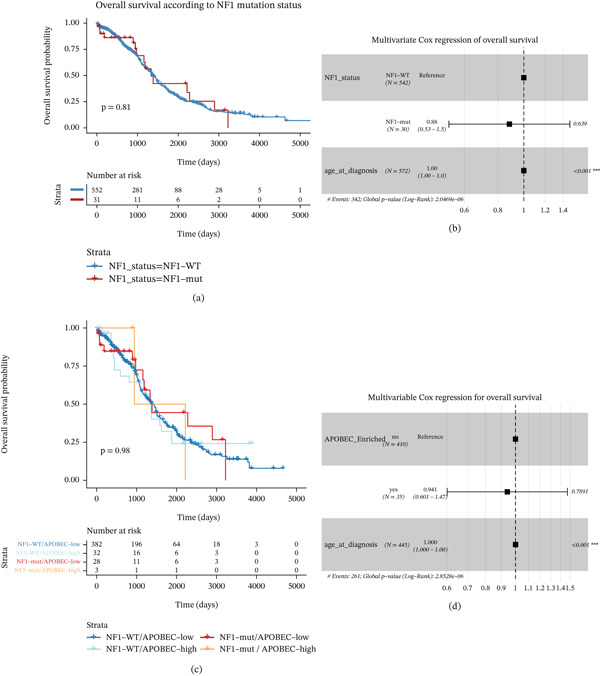
Clinical impact of NF1 mutation and APOBEC activity in ovarian cancer. (a) Kaplan–Meier analysis of overall survival comparing NF1‐mutant and NF1–wild‐type tumors shows no significant survival difference. (b) Multivariable Cox regression incorporating age at diagnosis demonstrates that NF1 mutation is not an independent predictor of overall survival. (c) APOBEC enrichment status is similarly not associated with survival after age adjustment. (d) Stratified Kaplan–Meier analysis combining NF1 mutation and APOBEC activity reveals no clinically meaningful survival divergence among molecular subgroups, showing no significant survival differences among molecular subgroups.

To evaluate the clinical relevance of NF1 alterations, we investigated the association between NF1 mutation status, APOBEC mutagenesis, and overall survival in the TCGA ovarian cancer cohort. Kaplan–Meier analysis revealed no significant difference in overall survival between NF1‐mutant and NF1–wild‐type tumors (Figure 4a), indicating that NF1 mutation alone does not provide prognostic stratification. Consistently, multivariable Cox regression incorporating age at diagnosis showed that NF1 mutation was not independently associated with survival (Figure 4b), whereas age remained significantly associated with outcome. Given the genomic context analyses of NF1 mutation and mutational processes, we further assessed whether APOBEC enrichment influenced patient outcomes. APOBEC enrichment was similarly not independently associated with survival after adjustment for age (Figure 4c). Stratified survival analysis combining NF1 mutation and APOBEC status likewise did not reveal significant survival differences across molecular subgroups (Figure 4d). Together, these findings indicate that NF1 mutation and APOBEC‐associated mutational processes define genomic context without translating into measurable differences in overall survival, supporting a model in which NF1 primarily modulates mutational architecture rather than acting as a prognostic determinant.

### 3.5. NF1 Mutation Is Associated With Coordinated Pathway‐Level Functional Reprogramming

To further elucidate the functional consequences of NF1 mutation beyond mutational co‐occurrence patterns, we performed pathway‐level and gene set–based analyses to infer downstream biological effects. Mutation effect–oriented pathway analysis revealed that NF1‐mutant tumors exhibited preferential enrichment of pathways related to DNA replication, cell cycle progression, RNA processing, and proteostasis, suggesting broad perturbation of fundamental cellular programs rather than isolated signaling alterations (Figure S1A). In parallel, gene set–based analysis using a curated panel of NF1‐associated and proliferation‐linked genes demonstrated significant coordinated activation patterns in NF1‐mutant ovarian tumors (Figure S1B). Notably, these gene sets were enriched for regulators of DNA synthesis, RNA splicing, protein turnover, and metabolic homeostasis, indicating that NF1 loss may act as a permissive genomic event facilitating global transcriptional and posttranscriptional reprogramming. Collectively, these findings suggest that NF1 mutation is associated with pathway‐level functional reprogramming rather than a single dominant oncogenic process.

### 3.6. NF1 Depletion Selectively Sensitizes Ovarian Cancer Cells to Replication Stress Without Affecting Basal Proliferation

**Figure 5:**
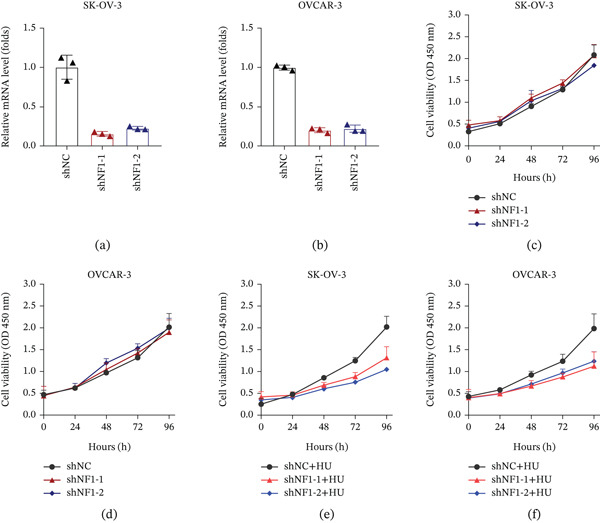
NF1 knockdown enhances replication stress sensitivity without altering basal proliferation in ovarian cancer cells. (a–b) Quantitative RT‐PCR showing efficient NF1 knockdown in (a) SK‐OV‐3 and (b) OVCAR‐3 cells expressing two independent NF1 shRNAs compared with control cells. (c–d) CCK‐8 assays showing comparable basal proliferation between NF1‐knockdown and control cells. (e–f) CCK‐8 assays following hydroxyurea treatment showing impaired growth of NF1‐deficient cells, indicating increased replication stress sensitivity. Data are presented as mean ± SEM from three independent experiments. Statistical significance was assessed using unpaired two‐tailed Student′s *t*‐tests (∗*p* < 0.05, ∗∗*p* < 0.01, ∗∗∗*p* < 0.001).

To experimentally interrogate the functional consequence of NF1 loss in ovarian cancer cells, we established stable NF1 knockdown models in SK‐OV‐3 and OVCAR‐3 cells using two independent shRNAs. Quantitative RT–PCR confirmed a consistent reduction of NF1 mRNA levels in both cell lines compared with negative control cells, validating efficient NF1 silencing (Figure 5a,b). We next examined whether NF1 depletion affects basal proliferation under unstressed conditions. CCK‐8 assays showed comparable growth kinetics between NF1‐deficient and control cells across all time points in both SK‐OV‐3 and OVCAR‐3 models, indicating that NF1 knockdown does not alter baseline proliferative capacity (Figure 5c,d). Given the genomic association between NF1 mutation and DNA damage–related pathways, we further assessed cellular responses to replication stress. Following HU treatment, NF1‐knockdown cells exhibited impaired growth compared with control cells in both ovarian cancer models, particularly at later time points (72–96 h). This phenotype was consistently observed with both shRNAs, indicating increased sensitivity of NF1‐deficient cells to replication stress (Figure 5e,f). Together, these findings indicate that NF1 loss does not affect basal proliferation but increases cellular sensitivity to replication stress.

### 3.7. NF1 Deficiency Exacerbates Replication Stress–Induced DNA Damage in Ovarian Cancer Cells

**Figure 6:**
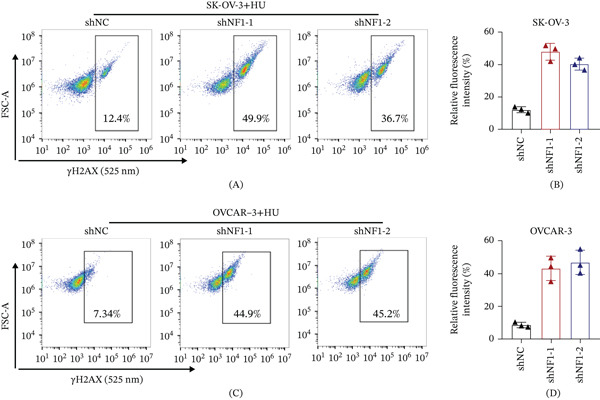
NF1 knockdown enhances replication stress–induced DNA damage in ovarian cancer cells. (A) Representative flow cytometry plots showing *γ*H2AX staining in SK‐OV‐3 cells following HU treatment. (B) Quantification of relative *γ*H2AX fluorescence intensity in SK‐OV‐3 cells. (C) Representative *γ*H2AX flow cytometry profiles of OVCAR‐3 cells. (D) Quantitative analysis of *γ*H2AX fluorescence intensity in OVCAR‐3 cells. Data are presented as mean ± SEM from three independent experiments. Statistical significance was assessed using unpaired two‐tailed Student′s *t*‐test (∗*p* < 0.05, ∗∗*p* < 0.01, ∗∗∗*p* < 0.001).

To determine whether NF1 loss influences DNA damage under replication stress, *γ*H2AX accumulation was examined in NF1‐depleted ovarian cancer cells following HU treatment. Flow cytometry revealed a clear increase in *γ*H2AX‐positive cell populations in SK‐OV‐3 cells expressing NF1‐targeting shRNAs compared with control cells after HU exposure (Figure 6A), accompanied by elevated relative *γ*H2AX fluorescence intensity (Figure 6B). Similar findings were observed in OVCAR‐3 cells, where NF1 knockdown increased both the proportion of *γ*H2AX‐positive cells (Figure 6C) and *γ*H2AX signal intensity under replication stress conditions (Figure 6D). These effects were consistently observed with two independent shRNAs across both cell lines, supporting an NF1‐dependent increase in DNA damage accumulation. Together, these data indicate that NF1 depletion does not alter basal proliferation but increases vulnerability to replication stress–induced DNA damage in ovarian cancer cells.

## 4. Discussion

This study integrates cohort‐scale somatic mutation profiling with targeted functional validation to define the biological meaning of NF1 loss in ovarian cancer. Rather than framing NF1 as a standalone prognostic biomarker, our analyses support a more nuanced model: NF1 truncating alterations occur within a specific mutational and DNA damage–oriented genomic context and are associated with increased stress‐contingent cellular vulnerability. This perspective is particularly relevant for ovarian cancer, a disease in which point mutation burden is generally constrained, whereas genome maintenance programs and mutational processes vary substantially across tumors [18, 42, 43]. By anchoring NF1 within these programs, our work provides an interpretable connection between somatic variant patterns in patient tumors and replication stress phenotypes in vitro.

A key observation is that NF1 alterations in ovarian cancer are predominantly truncating and consistent with loss of function, yet NF1‐mutant tumors do not exhibit a global increase in TMB. This result argues against a simplistic “NF1 as a hypermutator surrogate” interpretation and instead suggests that NF1 loss marks qualitative changes in mutational processes. Indeed, the substitution spectrum and APOBEC‐related analyses indicate that NF1 mutation is preferentially embedded in tumors with a distinct mutagenic milieu, even when the overall mutational quantity remains similar. Such dissociation between mutational burden and mutational process is biologically plausible: APOBEC activity and other context‐dependent mutagenic programs may reshape nucleotide substitution patterns and local sequence contexts without necessarily driving a large per‐megabase mutation count in all settings [21, 44, 45]. In ovarian cancer, where mutation counts are comparatively modest, capturing these qualitative process shifts may be more informative than relying on TMB alone.

Our data further place NF1 loss within a coordinated DDR alteration framework. The observed enrichment of comutations across DDR pathways—and the recurrent co‐occurrence of NF1 with key DDR genes—suggests that NF1 mutation is not an isolated event but part of a broader genome maintenance state. Importantly, we interpret this association conservatively: comutation enrichment does not prove that NF1 directly regulates DDR machinery, nor does it establish a single causal ordering. Instead, the most parsimonious explanation is that NF1 loss and DDR disruption cosegregate within a genomic ecosystem characterized by replication‐associated stress and compromised repair capacity [46–48]. This interpretation aligns with the concept that tumors may reach similar evolutionary endpoints through multiple, partially redundant destabilization routes—some centered on repair defects, others on mutagenic processes, and others on altered stress buffering—yielding correlated but not necessarily linear dependencies among mutated genes.

One of the most practical challenges in tumor genomics is interpreting recurrent mutations that do not obviously translate into clinical outcome. In this cohort, NF1 mutation was not an independent predictor of overall survival, and APOBEC enrichment similarly did not stratify survival in a robust manner. These findings are not contradictory to the biological associations described above; rather, they underscore that a mutation‐defined genomic state can be mechanistically meaningful while being clinically neutral in unselected populations. Overall survival in ovarian cancer is influenced by stage distribution, surgical cytoreduction, therapy regimens, HR deficiency status, and postprogression treatments, all of which can dilute the prognostic signal of any single gene alteration [49–51]. Moreover, mutations that shape tumor evolution and stress tolerance may act more strongly on treatment response or resistance trajectories than on baseline survival—an effect that is not fully captured by OS alone. Therefore, the absence of a strong prognostic association should be viewed as a boundary condition of interpretation, not as evidence against biological relevance [52].

To move beyond association, we performed functional validation designed to directly test the “stress‐context” hypothesis suggested by the genomic data. In two ovarian cancer cell models, NF1 knockdown did not alter basal proliferation, yet it markedly increased sensitivity to HU‐induced replication stress [53]. This pattern is informative: it argues that NF1 does not behave as a generic proliferation driver in these settings but instead functions as a buffering factor whose contribution becomes apparent when replication dynamics are perturbed. Consistent with this, NF1 deficiency enhanced *γ*H2AX accumulation under replication stress, indicating increased DNA damage burden or impaired damage resolution. Together, these data provide functional support for a model in which NF1 loss contributes to replication stress vulnerability.

These findings have potential translational implications, but they should be stated with appropriate restraint. The replication stress sensitivity observed here raises the hypothesis that NF1‐deficient ovarian tumors—especially those coaltered in DDR pathways—may exhibit enhanced dependence on replication stress response checkpoints. This could nominate therapeutic vulnerabilities to agents targeting replication stress signaling or DNA damage response pathways. However, clinical inference requires careful validation, including treatment‐annotated cohorts and direct pharmacologic testing in relevant models, particularly because ovarian cancer is already enriched for DDR‐related therapeutic strategies (e.g., PARP inhibition) and because the functional consequence of NF1 loss may differ across histologic and molecular subtypes. Accordingly, our results should be viewed as a mechanistic framework that prioritizes hypotheses for therapy‐oriented studies, rather than as immediate predictive biomarkers.

Several limitations warrant consideration. First, the cohort‐scale analyses are based on a single public dataset; independent validation in additional cohorts would strengthen generalizability, especially for comutation patterns and mutational process stratification. Second, APOBEC‐related signals were inferred computationally; orthogonal evaluation of signature contributions and integration with additional genomic layers (e.g., structural variants, copy‐number alterations, or HRD scores) could refine the genomic state definition. Third, our functional experiments focused on replication stress phenotypes in established cell lines; although the consistency across two lines and two independent shRNAs supports specificity, extending these observations to additional models, including patient‐derived systems, would clarify robustness. Finally, we did not delineate a detailed molecular mechanism, and we intentionally avoided protein‐level assays in this study design; future work can address mechanistic ordering among NF1 loss, DDR coalterations, and replication stress response dependencies.

## 5. Conclusion

In summary, our study reframes NF1 mutation in ovarian cancer as a marker of a DNA damage–oriented mutational and functional state rather than a simple prognostic determinant. By integrating mutational process analysis, DDR coalteration profiling, and stress‐contingent functional assays, we connect tumor‐level variant patterns to replication stress vulnerability. This provides an interpretable, biologically grounded view of NF1 loss in ovarian cancer and establishes a foundation for future studies aimed at refining mutation‐defined tumor states and exploiting associated DNA damage response liabilities.

## Author Contributions

S.H. and Q.W. designed and conceived this study. S.H., C.L., Z.L., T.L., and Q.W. drafted the manuscript. S.H., C.L., and Z.L. performed data analysis and visualization. Z.L. completed the in vitro experiments. Q.W. supervised and funded this study.

## Funding

This study was supported by Sichuan Province Science and Technology Department of foreign (border) high‐end talent introduction project 2023ZHYZ0009; Luzhou Science and Technology Department Applied Basic Research program (2022‐WYC‐196).

## Ethics Statement

This study analyzed publicly available, deidentified data from The Cancer Genome Atlas (TCGA). As all TCGA data are anonymized and publicly accessible, no additional institutional review board approval or informed consent was required for the analyses performed in this work. All experiments were conducted in vitro using established human cell lines; no primary human specimens or animals were involved.

## Conflicts of Interest

The authors declare no conflicts of interest.

## Supporting Information

Additional supporting information can be found online in the Supporting Information section.

## Supporting information


**Supporting Information 1.** Figure S1: Functional interpretation of NF1 mutation through pathway‐level and gene set–based analyses. (A) Mutation effect–oriented pathway analysis showing enrichment of fundamental cellular pathways associated with NF1 mutation, including DNA replication, cell cycle regulation, RNA processing, and proteostasis. (B) Gene set–based analysis using a curated panel of NF1‐associated and proliferation‐related genes showing increased enrichment scores in NF1‐mutant ovarian tumors.

**Supporting Information 2.** Figure S2.

## Data Availability

The somatic mutation and transcriptomic data analyzed in this study are publicly available from The Cancer Genome Atlas (TCGA) ovarian cancer (TCGA‐OV) project. All datasets used were obtained from open‐access repositories, and the specific sources and accession information are described in the Methods section. Experimental data generated in this study are included in the article and its supporting information. Additional data supporting the findings of this study are available from the corresponding author upon reasonable request.
