# Supplementary figures and images for: Integrative Genomic and Functional Analysis Reveals NF1 Loss as a Modifier of DNA Damage and Replication Stress Responses in Ovarian Cancer
Source: Hum Mutat. 2026 May 18;2026:9333284. doi: 10.1155/humu/9333284 (PMC13181431; doi:10.1155/humu/9333284)

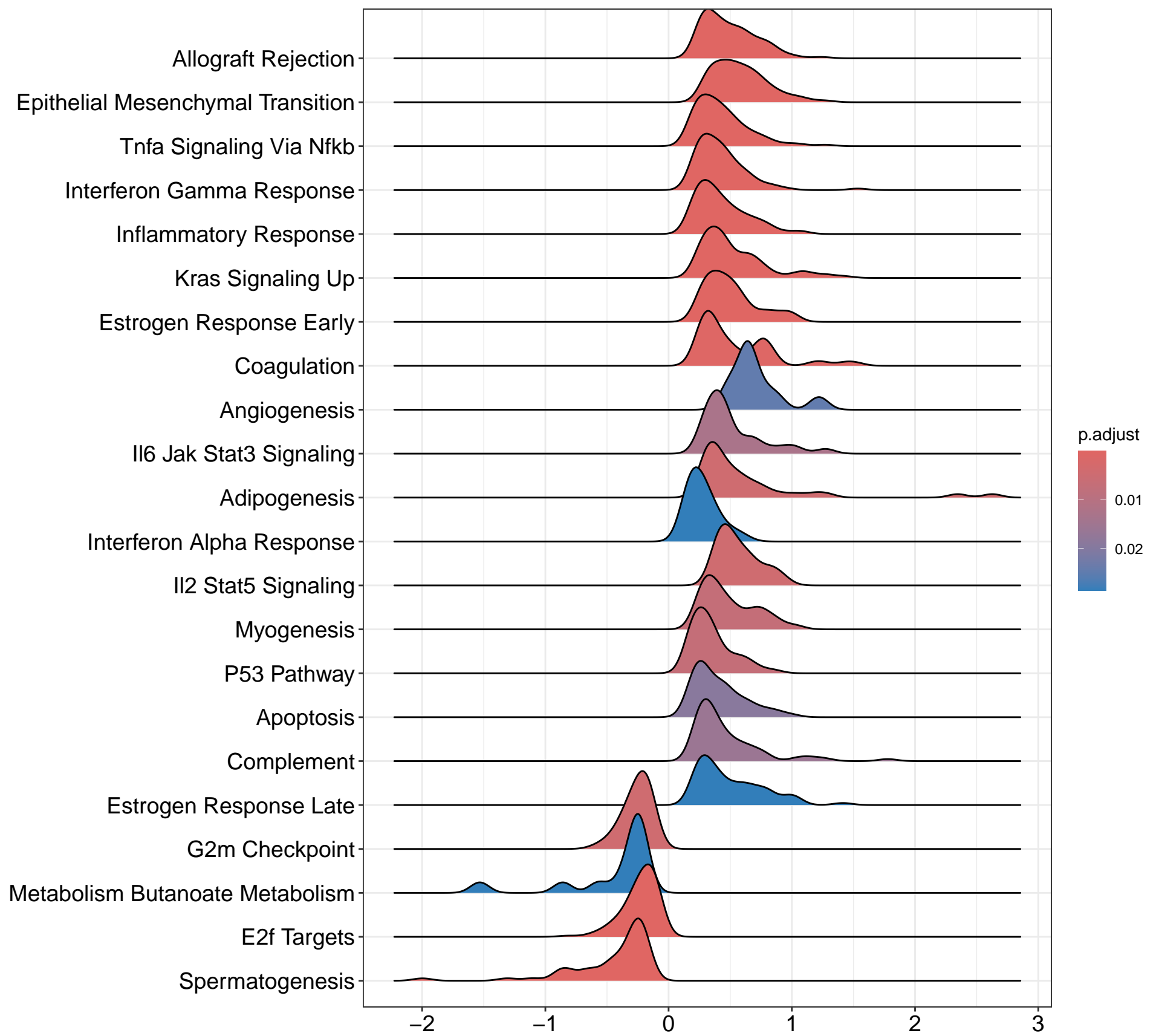

Supplement: Supplementary file 1 — Supporting Information 1. Figure S1: Functional interpretation of NF1 mutation through pathway‐level and gene set–based analyses. (A) Mutation effect–oriented pathway analysis showing enrichment of fundamental cellular pathways associated with NF1 mutation, including DNA replication, cell cycle regulation, RNA processing, and proteostasis. (B) Gene set–based analysis using a curated panel of NF1‐associated and proliferation‐related genes showing increased enrichment scores in NF1‐mutant ovarian tumors. [file HUMU-2026-9333284-s001.pdf]

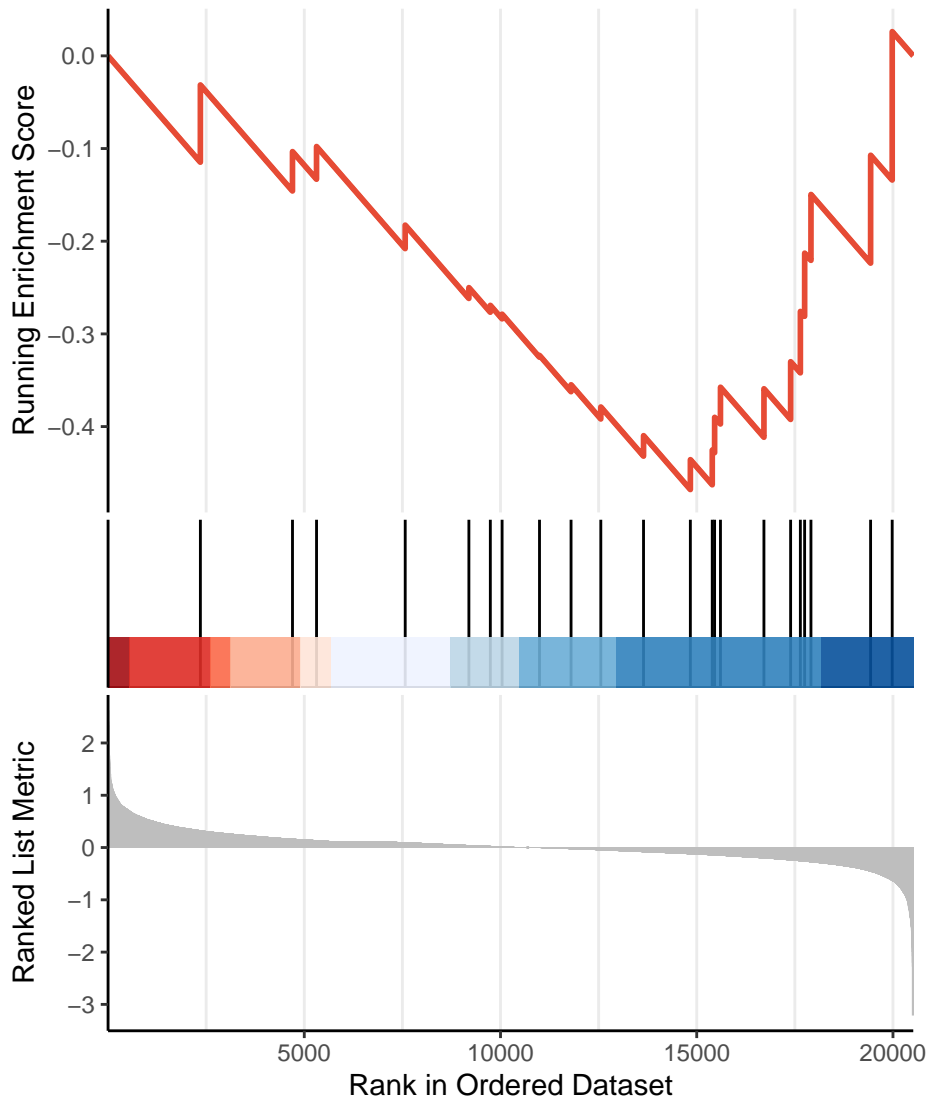

Supplement: Supplementary file 2 — Supporting Information 2. Figure S2. [file HUMU-2026-9333284-s002.pdf]
